# Benefits of Prehabilitation before Complex Aortic Surgery

**DOI:** 10.3390/jcm12113691

**Published:** 2023-05-26

**Authors:** Thomas Mesnard, Maxime Dubosq, Louis Pruvot, Richard Azzaoui, Benjamin O. Patterson, Jonathan Sobocinski

**Affiliations:** 1 Service de Chirurgie Vasculaire, Centre de l’Aorte, CHU Lille, 59000 Lille, France; 2 Univ. Lille, INSERM U1008—Advanced Drug Delivery Systems and Biomaterials, 59000 Lille, France; 3 Department of Vascular Surgery, University Hospital Southampton, Southampton SO16 6YD, UK

**Keywords:** prehabilitation, enhanced recovery after surgery, open aortic repair

## Abstract

The purpose of this narrative review was to detail and discuss the underlying principles and benefits of preoperative interventions addressing risk factors for perioperative adverse events in open aortic surgery (OAS). The term “complex aortic disease” encompasses juxta/pararenal aortic and thoraco-abdominal aneurysms, chronic aortic dissection and occlusive aorto-iliac pathology. Although endovascular surgery has been increasingly favored, OAS remains a durable option, but by necessity involves extensive surgical approaches and aortic cross-clamping and requires a trained multidisciplinary team. The physiological stress of OAS in a fragile and comorbid patient group mandates thoughtful preoperative risk assessment and the implementation of measures dedicated to improving outcomes. Cardiac and pulmonary complications are one of the most frequent adverse events following major OAS and their incidences are correlated to the patient’s functional status and previous comorbidities. Prehabilitation should be considered in patients with risk factors for pulmonary complications including advanced age, previous chronic obstructive pulmonary disease, and congestive heart failure with the aid of pulmonary function tests. It should also be combined with other measures to improve postoperative course and be included in the more general concept of enhanced recovery after surgery (ERAS). Although the current level of evidence regarding the effectiveness of ERAS in the setting of OAS remains low, an increasing body of literature has promoted its implementation in other specialties. Consequently, vascular teams should commit to improving the current evidence through studies to make ERAS the standard of care for OAS.

## 1. Introduction

Endovascular aortic repair has been shown to reduce postoperative morbidity and mortality compared to open aortic surgery (OAS) [1]. In the setting of aneurysmal or occlusive disease, it has even become the preferred treatment modality in patients with suitable anatomy [2,3,4]. These minimally invasive procedures allow for the treatment of high-risk patients who are not suitable for OAS by mitigating surgical stress. Despite this, OAS remains a proven treatment option in patients with extended occlusive aorto-iliac disease [5], complex aortic aneurysms defined as those with hostile infrarenal aortic neck features or extended aneurysms involving the renal and/or visceral arteries, or to salvage failure of previous endovascular treatment [6]. In these settings, OAS causes more physiological stress because of the invasive surgical approach and supra-renal cross-clamping requiring organ protection strategies [7]. These procedures are usually performed by experienced and high-volume teams and lend themselves to the implementation of measures to reduce associated morbidity. Nonetheless, the postoperative mortality rate remains as high as 4–8% in a contemporary series of OAS for complex AAA, mainly related to respiratory and cardiac events [8,9,10]. Analysis of a national registry showed that the proportion of patients treated by OAS for aorto-iliac occlusive disease declined by two thirds in the past 15 years, while no improvement in postoperative mortality was noted over the same period of time [11].

Prehabilitation refers to a combination of measures aiming to prepare patients for the physiological and psychological stress induced by surgery [12]. There is growing evidence that these programs confer benefits to patients [13] and promote enhanced recovery after surgery (ERAS). The ERAS evidence-based consensus statement based on this emerging knowledge and was initially described for colorectal surgery [14]. Based on various changes to overall care in this setting, similar guidelines were progressively integrated into other surgical specialties [15,16,17,18,19] and, more recently, for OAS [20]. This work aims to review the benefits of prehabilitation and ERAS in patients undergoing OAS.

## 2. Physiological Consequences of OAS for Complex Aortic Surgery

The physiological response that occurs because of surgical injury is referred to as the surgical stress response. It is mediated by an endocrine/inflammatory response and reduces tissue damage, prevents or combats infection, and initiates the healing process [21,22]. The intensity of the stress response is proportional to the surgical wound, internal organ manipulation and tissue dissection, and is correlated with postoperative adverse events [23].

OAS can be performed through a transperitoneal or a retroperitoneal approach when the disease remains limited to the abdominal aorta. The transperitoneal approach leads to a severe alteration in pulmonary mechanics [24] and bowel motility, resulting in higher rates of postoperative pneumonia and prolonged ileus [25]. Pulmonary complications are even more likely if a thoraco-abdominal exposure is performed, because of the detrimental effect on lung volume via alveolar collapse and pleural effusion, respiratory muscles and diaphragmatic dysfunction, all encouraging pulmonary infection [26]. Owing to the up to 50% increase in oxygen consumption after major abdominal surgery related to the elevated global oxygen demand, pulmonary complications may lead to serious consequences [27].

Aortic cross-clamping results in major hemodynamic changes including increased blood pressure, changes in afterload and cardiac output variability [28]. Unclamping results in reperfusion syndrome that triggers a systemic inflammatory response. The intensity of this effect is related to the level and time of clamping [29], and can result in multiple organ dysfunction, which can vary in intensity. In parallel, postoperative ischemia/reperfusion injuries are closely related to pre-existing organ impairment and the patient’s medical history [29].

Coagulopathy can occur during open vascular surgery and is multifactorial. The underlying mechanisms are complex and not fully understood [30]. Major intraoperative bleeding is typically the starting point, leading to the consumption of platelets and coagulation factors. The dilution of the coagulation factors induced by fluid resuscitation, including red cell transfusion, worsens the coagulation disorders [31]. Furthermore, acidosis occurs secondary to tissue hypoperfusion and impairs the coagulation process, resulting in delays in clot formation and reducing the clot strength [32]. Hypothermia may also contribute to acquired coagulopathy by decreasing platelets’ aggregability and adhesion.

Pre-existing platelet dysfunction secondary to chronic renal disease or antiplatelet therapy, in addition to the administration of heparin during vascular surgery, may contribute to exacerbations in the acquired coagulopathy consequences and challenge their management.

## 3. Preoperative Risk Assessment

These physiological consequences require physicians to conduct thorough risk stratification and optimise the treatment of pre-existing medical conditions. Cardiac complications account for up to 40% of adverse events after OAS [33]. Cardiac preoperative risk assessment usually includes recording the patient’s cardiovascular risk factors, and imaging or functional examinations including transthoracic echocardiography and a non-invasive stress testing. However, preoperative coronary revascularisation remains a matter of debate. According to the current guidelines, it should be considered only in patients with unstable coronary disease or in high-cardiac-risk patients [34].

To assess pulmonary risk, pulmonary function testing should not be used routinely, except in patients with previously diagnosed chronic obstructive pulmonary disease (COPD) or asthma [35]. Some authors advocate the liberal use of pulmonary function testing to detect undiagnosed COPD, allowing for a better selection of patients that would benefit from the implementation of medical therapy and prehabilitation [34]. Although pulmonary function testing can highlight undiagnosed pulmonary disease such as COPD, obesity hypoventilation syndrome or pulmonary artery hypertension, the results are poorly correlated with the patient’s functional status [36]. It is acknowledged that good functional status is correlated with a better prognosis, even in patients with numerous risk factors [37]. Therefore, other tools should be used for the identification of patients who may benefit the most from prehabilitation programs. Functional capacity is expressed in metabolic equivalents (MET), which reflect the ability to perform and cope with activities of daily living and the physiological capacity to increase cardiac output to meet elevated post-surgical oxygen demands [26]. Some authors have proposed the use of cardiopulmonary exercise testing as an objective measure of cardiorespiratory performance, estimated, among other measures, by the peak oxygen consumption during exercise (VO_2_-peak) [38]. This tool provides an accurate and reproducible assessment of functional status but requires resources and expertise that might not be available in all centres [36]. The MET can be subjectively estimated based on the capacity to perform certain tasks. The ability to climb two flights of stairs or run a short distance without symptoms corresponds to ≥4 MET, or a moderate activity level [39]. It has been suggested that these patients could proceed to surgery without further cardiac work-up [36]. However, the prognostic accuracy of this biased evaluation has been questioned [40]. Subjective measures have been compared to three alternative methods (including a validated standardised questionnaire (Duke Activity Status Index [DASI]), peak oxygen consumption measured during cardiopulmonary exercise testing and preoperative serum N-terminal pro-B-type natriuretic peptide (NT pro-BNP) concentrations) in a prospective multicentre cohort study of 1401 patients undergoing elective major non-cardiac surgery [41]. While both DASI questionnaire and NT pro-BNP levels predicted the 30-day mortality, and the peak oxygen consumption predicted postoperative complications, the subjective method did not predict any outcomes reliably.

Although chronic renal insufficiency is a surrogate marker for all-cause postoperative mortality after OAS, there are currently no effective strategies besides hydration to prevent post-operative kidney injury. However, preoperative serum creatinine concentration should be measured, and patients referred to specialist renal services, when creatinine clearance is <60 mL/min/1.73 m^2^. [34]

Malnutrition, estimated by a serum albumin level < 30–35 g/L, negatively correlates with pulmonary complications after non-cardiac major surgery [35,42], and surgical site infection in general surgery [43]. In the context of OAS, a multi-institutional study pooled analysis of 4956 patients undergoing OAS for AAA highlighted a detrimental severity-dependent association between preoperative serum albumin level and outcomes (30-day mortality, pulmonary complications, and length of stay) [44]. This study emphasized the need for the routine screening of preoperative serum albumin, allowing for poor nutritional status to be adequately corrected. Current guidelines also recommend that nutritional risk screening includes a record of body mass index (BMI), the percentage of weight loss within three months, and documentation of food intake [45].

The diagnosis and management of preoperative anaemia is of relevance before major vascular surgery, taking into consideration the high risk of major blood loss and the high cardiovascular risk of the involved population. The World Health Organization (WHO) defines anaemia as a hemoglobin (Hb) count <12.0 g/dL in female and <13.0 g/dL in male subjects [46]. Teams should adequately address preoperative anaemia in all patients undergoing OAS and define a transfusion plan. To allow for adequate time to optimize erythrocyte mass, laboratory testing should be completed 4–6 weeks prior to the operative date [47,48]. Frailty is a multifactorial state of impaired functional reserve and decreased resistance to stressors, and better predicts surgical risk compared to age itself in elderly patients. Frailty is also associated with delayed recovery and decline function after major surgery. [49] Thus, frailty screening appears to be of particular interest to implement interventions aiming to mitigate these risks [50]. Comprehensive geriatric assessment is the gold standard for frailty assessment but is limited by medical resources and is not relevant in all elderly surgical patients. Thus, numerous screening scores exist, including the Clinical Frailty Scale. This is a quick nine-point scale that does not require physical performance test. The feasibility of the test is particularly important when considering the limited time attributed to outpatient care [51]. Among selected patients, comprehensive geriatric assessment enables the identification and remediation of contributors to frailty (physical performance, nutrition, cognition, polypharmacy, and mental health) [51]. However, the beneficial impact of dedicated prehabilitation programs on frail patients still needs to be investigated [52].

## 4. Management of Postoperative Risk with an Active Preoperative Program

### 4.1. Prehabilitation

It stands to reason that patients with a higher functional capacity are more likely to have an uneventful postoperative course [37,41,53]. Thus, prehabilitation presents the opportunity to prevent postoperative adverse events by improving patients’ functional capacity through a combination of measures. Prehabilitation also aims to promote the enhanced and full recovery of preoperative functional capacity by increasing physiological reserve. Prehabilitation has evolved from unimodal intervention (exercise or nutrition alone) to a multimodal and multidisciplinary preoperative approach [54]. Despite the lack of a consistent definition, prehabilitation is known as a combination of exercise interventions aiming to improve conditioning before surgery. These programs usually include aerobic exercises (cycling and walking), resistance training, and specific deep-breathing training and exercises. Multimodal prehabilitation encompasses this exercise training, along with dietary interventions, psychological support, smoking and alcohol cessation, and medical optimization.

Primarily, physicians encourage compliance through counselling and education. In the context of upper abdominal surgery, the benefit of a 30 min preoperative physiotherapy session including education and breathing exercise training has been demonstrated in a randomized control trial (RCT) [55]. The absolute reduction risk of postoperative pulmonary complications was as high as 15% in the intervention group. Patients should commit to smoking cessation through a dedicated road map in connection with addiction specialists and psychologists [56]. This decreases the rate of pulmonary complications by more than 20% if initiated for 4 weeks before surgery [57] and reduces the risk of postoperative infections [58].

Therefore, the current concept of prehabilitation encompasses multifaceted exercise programs that aim to improve cardiovascular function and enhance thoracic muscle strength. In particular, these programs have been studied in the context of thoracic and cardiac surgery and have proven benefits in terms of preoperative exercise capacity, reduction in the number of days before chest drain removal, postoperative complications, and total length of hospital stay [59,60]. Programs are usually delivered in an outpatient setting for from 6 to 8 weeks, but shortened durations are possible to fit within surgical time frames without compromising its advantages [60,61]. Despite the absence of a standardized protocol, the impact of a prehabilitation program in terms of functional capacity improvement can be indirectly assessed by measuring markers of respiratory muscle strength (maximum inspiratory pressure) and distance walked over a specific time (six-minute walk test). Prehabilitation before elective AAA open repair has been examined in two RCTs [62,63]. The oldest one was a pilot study published in 2008 and focused on the impact of preoperative inspiratory muscle training on the incidence of postoperative atelectasis. Only 20 patients were enrolled, but the incidence and duration of postoperative atelectasis was reduced [63]. The second RCT was published in 2016 and enrolled 124 patients. The six-week prehabilitation program significantly decreased the incidence of a composite criterion including cardiac, pulmonary, and renal complications (22.6% vs. 41.9%), and hospital stay by one day (7 vs. 8 days). Additional benefits were noted in patients with chronic arterial occlusive disease because of their generalized atherosclerosis and high risk of cardiovascular events [64]. Despite this, physiological deconditioning related to limitations to daily living activities and non-healing wounds may hamper the feasibility of exercise-based conditioning intervention before surgery in patients suffering from peripheral artery disease.

Preoperative nutritional support should be considered in those with hypoalbuminemia, particularly if severe (<2.8 g/dL). Current guidelines on clinical nutrition in surgery advocate nutritional counselling and the oral provision of nutritional support (oral nutritional complements, enteral nutrition) for a preoperative period of 7–14 days [45]. There is no available evidence supporting the implementation of immune enhancing (IE) nutrition over standard nutrition in OAS. In the context of peripheral atherosclerotic occlusive disease, malnutrition is frequent, is an additional risk factor for the severity of vascular disease [65,66], and is associated with impaired functional status [67]. Additionally, because of the extensive nature of the atherosclerotic disease or in cases of paravisceral “coral reef” [68], patients may have concomitant visceral arteries occlusive lesions and experience weight loss even without the typical sign of post-prandial abdominal pain [69] encountered in chronic mesenteric ischemia. These patients represent a more fragile population of patients, with severe co-morbidities and a higher cardiovascular risk [70]. Aside from cases requiring prompt arterial reconstruction, the tolerance of preoperative enteral feeding and nutritional support should be carefully monitored; otherwise, a parenteral nutritional support may be preferred [69].

In addition to the nutritional and exercise aspects of the prehabilitation, surgical teams should not underestimate the psychological consequences of the surgery itself. Preoperative psychological stress or anxiety has been associated with poorer surgical outcomes and longer hospital stay [71]. Furthermore, psychological health is closely related to functional status, as depressed patients are generally less physically active. Preoperative psychological intervention has been investigated in the settings of oncologic and cardiac surgeries as a tool to mitigate the serious distress patients face in the weeks leading up to surgery [72,73]. Similarly, abdominal aortic surgery can lead to substantial psychiatric morbidity, such as major depressive and posttraumatic stress disorders, and may affect up to one-third of patients [74]. The psychological consequences of OAS have been investigated in a prospective study including 216 patients undergoing elective aneurysm repair. In this study, postoperative psychiatric disorders (mood or anxiety) were more likely after OSR compared to EVAR or conservative treatment and in patients with a history of major depression [75]. The benefit of a brief cognitive behavioural intervention before coronary artery bypass graft surgery has been investigated in a randomized controlled trial [73]. Patients from the intervention group were less likely to present depressive or anxiety symptoms, had an improved quality of life score and a shorter hospital length of stay. To the best of our knowledge, the impact of such psychological prehabilitation programs has not been investigated in the context of OAS.

### 4.2. Enhanced Recovery Pathways

ERAS is a perioperative care pathway aiming to accelerate patients’ recovery and discharge while reducing postoperative complications. Based on RCTs, fast-track surgery programs have become the standard of care in colorectal surgery [57,76]. The benefits in the setting of AAA open repair were also evaluated in an RCT in the late 2000s [77]. This study showed encouraging results, with decreased postoperative assisted mechanical ventilation, fewer medical complications and faster recovery of gastrointestinal function. A recently published meta-analysis found benefits of ERAS in OAS in terms of hospital stay and postoperative complications, while no difference was found in 30-day mortality [78].

There is no available standardized ERAS protocol, but it is acknowledged that the positive influence on outcomes mainly depends on a combination of measures, with the isolated effect of individual elements being less important [79]. Integral components that should be considered and protocoled in all enhanced recovery pathways are fasting and carbohydrate loading, multimodal analgesia, prevention of postoperative nausea and vomiting, patient warming, anaesthetic protocols, postoperative fluid management, catheter/drain removal, early mobilization, and chest physiotherapy [20,78].

Patient blood management (PBM) is a recently defined multidisciplinary multimodal approach to limit the use and need of allogenic blood transfusions in at-risk patients. Preoperative anaemia is present in as many as 40% of surgical patients [80] and can have multiple aetiologies, including iron deficiency, occult blood loss, chronic kidney disease, cancer, or chronic inflammatory state. Intuitively, anaemia should be associated with an increased incidence of postoperative adverse events given the first principles of physiology regarding the balance between organ oxygen supply and demand. In addition, patients with vascular conditions are at higher risk because of the disseminated nature of the atherosclerotic disease. An increased risk of postoperative major adverse cardiac events was found in patients with preoperative anaemia in a vascular surgery population, with a severity-dependant association [81]. A meta-analysis pooled the results of 949,445 patients from 24 studies undergoing all types of surgery and highlighted that preoperative anaemia tripled the risk of in-hospital mortality [82]. Nonetheless, a multicentre study demonstrated that transfusion itself was an independent predictor of postoperative morbidity and mortality in 2946 patients undergoing major vascular surgery [83]. The uncertainty of whether allogeneic red cell transfusion is associated with harm or benefit in anaemic patients remains unsolved [84]. Transfusion exposes the patient to a higher risk of infections including surgical-site infections and pneumonia [85,86,87] related to its immunosuppressive effects [46], and additionally incurs considerable costs [88].

From this perspective, the goal of PBM is to reduce transfusion-related adverse events while decreasing costs. One of its principles relies on the optimization of red blood cell mass and the perioperative use of PBM has been increasingly employed to improve patient outcomes [89].

Iron deficiency is known as one of the most prominent causes of anaemia [90] and should be corrected postoperatively. Either oral or intravenous iron can be used. Although more expensive, intravenous iron supplementation therapy has the advantage of causing fewer gastrointestinal side effects, which are known to hamper the compliance of oral iron and its absorption [91]. In addition, intravenous iron is more effective in raising the haemoglobin level in a shorter period of time compared to oral intake, which represents an advantage if there are preoperative time constraints. Previous studies highlighted the multiple advantages of preoperative intravenous iron protocols, including a decrease in the need for blood transfusion, less postoperative acute kidney injury and infections, and a reduction in the hospital length of stay [92,93]. Recombinant human erythropoietin can be an adjunct to iron supplementation therapy in specific patients, such as those with end-stage chronic kidney disease [94].

In addition to the diagnosis and management of preoperative anaemia, teams should define a transfusion plan for hemodynamically stable patients based on patient’s comorbidities and clinical tolerance. Restrictive transfusion thresholds (<8 g/dL) in asymptomatic patients are now recommended over liberal transfusion thresholds owing to the similar postoperative mortality rates [95]. Cell salvage is commonly advocated in surgery with anticipated blood loss of over 1000 mL, with the aim of reducing the need for allogenic transfusion, and is widely used in the context of OAS [96,97]. Cell salvage relies on the use of blood salvage devices that collect, heparinize and centrifugate shed blood to separate red cells from plasma. The bowl is then washed with saline to remove fat particles and haemolytic fragments [98]. Although the centrifugation and washing processes drastically reduces the number of white blood cells and inflammatory mediators [99], a systemic pro-inflammatory response persists after re-transfusion [100,101]. The other drawbacks of cell salvage are the risk of dilutional coagulopathy and the risk of renal failure in cases of massive autologous transfusion [96].

## 5. Limitations to Application

A previous review investigating the beneficial effects of preoperative exercise therapy in patients undergoing AAA surgery was published in 2015 [12]. Only five studies were included, and the benefits of such programs were unclear. A meta-analysis published in 2022 pooled the results of only 10 studies (including two RCTs) with 709 patients, focusing on the impact of ERAS in the setting of OAS (Table 1) [78]. This highlights the tempered enthusiasm of the vascular community for ERAS in comparison to cardio-thoracic or general surgery. This may be partly explained by the shift toward minimally invasive approaches with endovascular techniques. The value of ERAS in endovascular aortic repair is less obvious, although it can be argued that preoperative management and treatment optimization are always valuable for patients’ health.

The attendance and completion of pre-operative exercise programs is known to be poor [102], and somewhat limits increases in experience. The implementation of ERAS confers additional complexity to the patient pathway, and alterations in traditional practices may be met with some reluctance by physicians averse to change. One last and potentially major advantage of ERAS is that it can generate additional income for the Hospital while limiting resource consumption and readmission rates [103,104].

## 6. Conclusions

OAS for complex aortic surgery involves a multidisciplinary team and advanced perioperative interventions to decrease morbidity and mortality. Although the benefit of prehabilitation and ERAS seem likely to be more pronounced in the context of OAS, there is enough available data supporting their extended use for them to become the standard of care in all aortic surgery. Based on favourable results from other disciplines, there is enough evidence to support the implementation of ERAS in OAS, as such interventions appear to reduce adverse postoperative events and hospital length of stay while being cost-effective. ERAS needs to be further investigated through prospective or randomized control studies. The identified limiting factors may slow its more general applicability.

## Figures and Tables

**Table 1:** Results of randomized control trials (RCT) and meta-analysis investigating prehabilitation or enhanced recovery programs in open aortic surgery.

Study (Design)	Study Population	Intervention	Main Outcomes	Results
Prehabilitation				Interv. vs. Control Group
Dronkers et al., 2008 [63] (RCT)	N = 20 Mean age 70 ± 6 years (intervention) vs. 59 ± 6 years (control)	Inspiratory muscle training program (≥2 weeks before surgery)	Postoperative pulmonary atelectasis	3/10 vs. 8/10; *p* = 0.07
Barakat et al., 2016 [62] (RCT)	N= 120 (including 46 EVAR) Male sex = 93% Mean age 73 ± 7 years	6 weeks of preoperative supervised exercise	Composite endpoint including cardiac, pulmonary, and renal complications LOS	23% vs. 42%; *p* = 0.021 7 (5, 9) vs. 8 (6, 12.3) days; *p*= 0.025
ERAS				
Muehling et al., 2009 [77] (RCT)	N = 99 Median age 67 (40–81)	ERAS protocol	Surgical complications and reoperations Medical complications	10% vs. 8%; *p* = 0.741 16% vs. 36%; *p* = 0.039
Docherty et al., 2022 [78] (Meta-analysis)	Control group: N = 930 Intervention group: N = 709 10 studies included	Various ERAS programs	Postoperative complications LOS 30-day mortality	Ref = Interv. group OR = 0.38 (0.22, 0.65); *p* = < 0.001 −3.18 days (−5.01, −1.35); *p* < 0.001 No difference (*p* = 0.92)

ERAS: enhanced recovery after surgery; EVAR: endovascular aneurysm repair; Interv.: Intervention; LOS: length of stay; OR: odds ratio; RCT: randomized control trial; Ref: reference.

## Data Availability

Not applicable.
